# Immunotherapy in Anal Cancer

**DOI:** 10.3390/curroncol30050343

**Published:** 2023-04-27

**Authors:** Natasha Dhawan, Muhammad Z. Afzal, Manik Amin

**Affiliations:** Department of Medical Oncology, Dartmouth Cancer Center, Lebanon, NH 03756, USA; natasha.dhawan@hitchcock.org (N.D.); muhammad.afzal@hitchcock.org (M.Z.A.)

**Keywords:** immunotherapy, anal squamous cell carcinoma, HPV

## Abstract

The incidence and mortality of squamous cell carcinoma of the anus has been gradually increasing globally over the last few decades. The evolution of different modalities, including immunotherapies, has changed the treatment paradigm of metastatic anal cancers. Chemotherapy, radiation therapy, and immune-modulating therapies form the backbone of treatment of anal cancer in various stages. Most anal cancers are linked to high-risk human papilloma virus (HPV) infections. HPV oncoproteins E6 and E7 are responsible for an anti-tumor immune response triggering the recruitment of tumor-infiltrating lymphocytes. This has led to the development and utilization of immunotherapy in anal cancers. Current research in anal cancer is moving forward to discover ways to incorporate immunotherapy in the treatment sequencing in various stages of anal cancers. Immune checkpoint inhibitors alone or in combination, adoptive cell therapy, and vaccines are the areas of active investigations in anal cancer in both locally advanced and metastatic settings. Immunomodulating properties of non-immunotherapies are incorporated to enhance immune checkpoint inhibitors’ effectiveness in some of the clinical trials. The aim of this review is to summarize the potential role of immunotherapy in anal squamous cell cancers and future directions.

## 1. Introduction

Cancers of the anus are rare and account for 2.8% of all gastrointestinal malignancies [1]. The most common histologies include: squamous cell carcinoma of the anus (SCCA), adenocarcinoma, and neuroendocrine tumors, with SCCA accounting for almost 90% of all cases in the United States. The American Cancer Society predicts 9760 (3180 in men and 6580 in women) new cases and 1870 (860 in women and 1010 in men) deaths related to anal cancer in 2023 [1,2]. Though the prevalence of SCCA is low, the National Cancer Institute’s Surveillance, Epidemiology, and End Results (SEER) database from 2001–2015 revealed the incidence has increased 2.7% per year and mortality increased by 1.9% annually, especially in young black men and older women between the ages of 60 and 69 [3]. The relative 5-year survival rate is 82% for localized anal cancer, 66% with regional spread, and 35% in the metastatic setting [4]. Due to the overwhelming proportion of SCCA, this review will discuss the role of immunotherapy specifically in SCCA.

## 2. Immunogenicity in Anal Cancer

**Figure 1:**
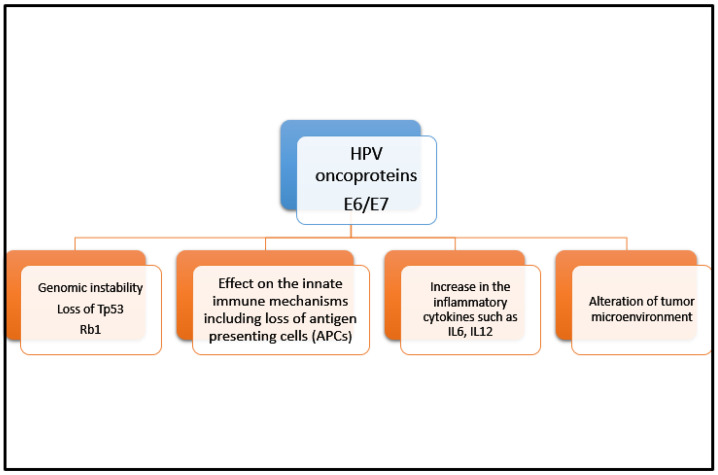
Immunogenicity of HPV oncoproteins. This figure highlights pathways whereby HPV oncoproteins E6 and E7 can induce an anti-tumor immune response triggering the recruitment of tumor-infiltrating lymphocytes. There is a complex interplay between multiple mechanisms including genomic instability, innate immune system impairment, inflammatory cytokine increase, and tumor microenvironment alterations.

Individuals with an immunocompromised state are at higher risk of developing cancer such as anal cancer [5]. Risk factors include infection with HPV, HIV infection, increased number of sexual partners, anal intercourse, chronic immunosuppressive states such as in transplant recipients and those with autoimmune conditions, chronic corticosteroid use, and cigarette smoking [6,7,8]. Human papilloma virus (HPV) infections, especially HPV 16 and 18 which are high-risk strains of HPV, account for 90% of patients with SCCA [5,6,7,8,9]. However, SCCA does not develop in most patients with HPV infection. In fact, most individuals can clear their HPV infection spontaneously without ever developing anal intraepithelial neoplasia (AIN) or more invasive malignancies. There are additional risk factors that predispose some individuals to have persistent HPV infections. For example, individuals who are co-infected with HPV and HIV, and may subsequently develop AIDS, are at higher risk of developing anal cancer [9]. This association further supports that the immunogenicity of anal cancer is triggered by significant immunosuppressive states in the setting of HIV/AIDS, enabling the HPV infection to integrate itself into the cell DNA for immune evasion. It is also reported there is an upregulation of immune modulators such as programmed death cell ligand-1 (PD-L1) and transforming growth factor beta (TGFβ) that further adds to immune evasion [10,11]. Moreover, E6 and E7 oncoproteins associated with HPV 16 result in recruitment of tumor infiltrating leukocytes (TILs) in the tumor microenvironment (TME). E6 binds to the p53 tumor-suppressor gene and E7 binds to phosphorylated retinoblastoma protein (Rb) leading to inhibition of apoptosis and uncontrolled cell proliferation. Those with an immunocompromised state are unable to prevent this proliferation of oncoproteins in the host cell. This infiltration of TILs results in interferon-γ secretion that is also associated with increasing PD-L1 expression/upregulation within TME [12,13]. Figure 1 depicts aspects of immunogenicity of HPV oncoproteins.

## 3. Role of Immunotherapy in Non-Metastatic (Early-Stage) Anal Cancer

The current standard of care approach to non-metastatic anal cancer is chemoradiation. Local excision is reserved for a select chemoradiation refractory population. Chemoradiation involves 5-fluorouracil (5-FU) and mitomycin C with concurrent radiation, though 5-FU and cisplatin may be considered. These regimens have demonstrated a complete response rate in the realm of 70–80% with a higher rate of sphincter preservation compared with upfront surgery. Unfortunately, patients who initially have a higher T stage (T3, T4) or node positive disease may present with local recurrence or persistent disease after chemoradiation, and salvage surgery may be pursued.

Currently, there are no established guidelines or indications for the use of immunotherapy with localized anal cancer. However, given the success of checkpoint inhibitors in the metastatic SCCA setting, there are trials underway evaluating the potential benefit of immunotherapy in this setting. The ECOG-ACRIN 2165 trial is currently evaluating the disease-free survival of patients with high-risk, localized anal cancer who receive nivolumab versus observation after definitive chemoradiation therapy [14]. Additionally, the German Anal Cancer Group is conducting the RADIANCE trial, which is to evaluate the disease-free survival of patients undergoing concurrent chemoradiation with durvalumab compared with those who receive chemoradiation alone. The results of both trials are still awaited at the time of this review [15]. The immunotherapy trials in non-metastatic settings are summarized in Table 1.

## 4. Role of Immunotherapy in Locally Advanced and Metastatic Anal Cancer

Metastatic SCCA represents 10–30% of anal cancers and the associated 5-year survival is approximately 35% [16]. Platinum-based chemotherapy in combination with fluoropyrimidine, Taxol, and anthracycline, such as mitomycin regimens, have been used in the past for the treatment of metastatic SCCA with the response rate ranging from 15% to 60% in various studies [17,18,19,20,21]. However, in 2018, due to lack of consensus on the optimal first-line regimen for metastatic anal cancer, a multicenter phase II “InterAACT” trial compared carboplatin/paclitaxel combination prospectively with cisplatin/5-FU combination in metastatic SCCA in first-line settings. The response rates were 59% and 57.1%, respectively, whereas the median overall survival (OS) rates were 20 months and 12.3 months, respectively (HR 2.0, *p* = 0.01) [22]. Currently, based on these results, carboplatin/paclitaxel is the preferred first-line regimen in metastatic SCCA.

**Figure 2:**
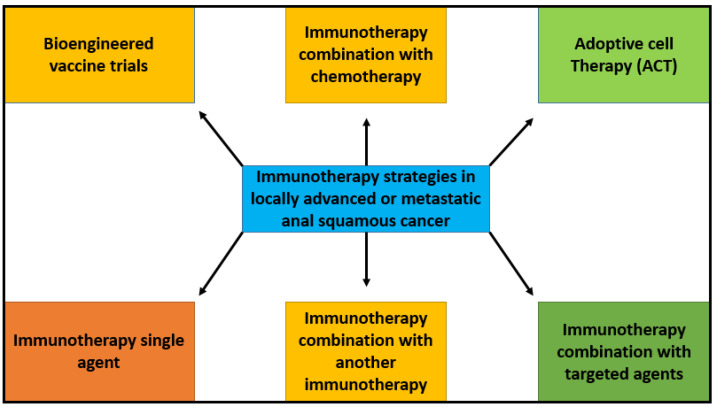
Immunotherapy strategies in metastatic anal squamous cancer. Schematic summary of potential strategies for the use of immunotherapy in locally advanced or metastatic anal squamous cell cancer. These strategies include chemoimmunotherapy, immunotherapy as monotherapy, combination immunotherapies, adoptive cell therapy, vaccine therapy, and immunotherapy combined with targeted therapies. Targeting different and multiple mechanisms of immune response increase potential treatment options and outcomes for patients.

Given the immunogenicity potential of HPV in squamous cell anal carcinoma, increasing PD-L1 expression and TGFβ upregulation through immunotherapy is an active area of interest in SCCA. Immunotherapy is being studied as a monotherapy as well as in combination with chemotherapy and targeted agents in SCCA. Figure 2 depicts various strategies of immunotherapy application in anal cancer. There are several early- and late-phase clinical trials reported in anal cancer.

## 5. Immune Checkpoint Inhibitors (ICI) as Monotherapy

In HPV-associated malignancies, ICI as a monotherapy is reported to have a response rate of 10–25% [23]. Nivolumab is the PD-1 blocker and was one of the first checkpoint inhibitors to be investigated in SCCA. Moris et al. reported a single-arm phase II trial of nivolumab in 37 pretreated refractory recurrent locally advanced or metastatic SCCA patients. The trial demonstrated an objective response rate (ORR) of 24% with two patients achieving complete response (CR). The longest duration of response was 10.4 months, median progression-free survival (PFS) was 4.1 months, and the median overall survival (OS) was 11.5 months. The trial enrolled two HIV-positive patients who had an optimal CD4-positive count and were on stable anti-retroviral therapy. Overall, nivolumab was very well tolerated; five patients experienced grade 3 immune-related adverse events and no patient experienced grade 4 adverse events (NCT02314169) [23].

Another PD-1 blocker, pembrolizumab, was also studied in SCCA. KEYNOTE-158 was a non-randomized multicohort, phase II clinical trial evaluating previously treated recurrent locally advanced or metastatic SCCA patients. The ORR was 11% with six patients achieving CR. In patients with CPS ≥ 1, the response rate was 15% compared with only 1% in patients with CPS < 1. Median PFS was two months and median OS was 11.9 months (NCT02628067) [24]. KEYNOTE-028 (NCT02054806) was a phase I trial assessing the safety and efficacy of pembrolizumab 10 mg/kg in solid tumor with PD-L1 ≥ 1%. In subgroup analysis on advanced-stage SCCA patients, among 24 patients with SCCA histology, four patients had a confirmed partial response (17%) and 10 patients had stable disease (42%), with a disease control rate (DCR) of 58%. Overall, 16 (64%) patients experienced treatment-related adverse events and there were no treatment-related deaths [12]. Later, in a pooled analysis of both of these KEYNOTE studies that included 137 patients of advanced SCCA, 73% patients had PD-L1 tumors. The ORR was 14% in PD-L1-positive patients and 10.9% in the overall patient population. Among responders, the median duration of response was ≥24 months. In the overall population, the median PFS and mOS were 2.1 months and 11.7 months, respectively [25].

Retifanlimab is a PD-1 blocker monoclonal antibody investigated in an open-label multicenter phase II trial on SCCA patients who progressed on platinum-based chemotherapy (POD1UM-202). In this trial, 94 patients were enrolled and followed for a median duration of 7.1 months. Retifanlimab demonstrated an ORR of 13.8% with a CR of 1.1% and PR of 12%. The response occurred regardless of PD-1 expression. Stable disease was noted in 35.1% patients with a DCR of 48.9%. The median PFS was 2.3 months and median OS was 10.1 months [26].

Avelumab is another PD-L1 blocker humanized monoclonal antibody, also investigated in advanced-stage/recurrent previously treated SCCA. CARCAS is an open label, multicenter, randomized phase II clinical trial (NCT03944252) with or without cetuximab. In the avelumab arm, the ORR was 10%, three patients achieved PR, the DCR was 50%, the median PFS was 2.0 months, and the OS was 13.9 months [27].

ICI monotherapy has demonstrated a modest response rate of 10–14% in all reported trials; however, a durable response was observed in responders, lasting over 2 years, as reported above [23,24,25,26,27]. Identifying patients who would achieve a durable response to immunotherapy is an active area of research in all types of malignancies.

## 6. Immune Checkpoint Inhibitors (ICIs) in Combinations

ICIs are used in various combinations in advanced-stage anal cancer. Endothelial growth factor receptor inhibitors (EGFRis) such as bevacizumab and cetuximab are both studied in combination with immune checkpoint inhibitors. EGFRis have immune modulatory effects and have been shown to increase CD3+ T-cells, CD8+ T-cells, and natural killer (NK) cells in the tumor microenvironment, whereas the regulatory T-cells numbers gradually decreases following treatment with EGFRis such as cetuximab. This recruitment of immune modulators has shown to increase responsiveness to ICIs when used in combination with EGFRis in various cancers such as lung and colorectal cancer [28,29]. Cetuximab was the first EGFRi studied in combination with PD-L1 inhibitor avelumab in a phase II CARACAS trial (NCT03944252), an open-label, randomized multicenter trial. This trial consisted of one arm with avelumab monotherapy and a second arm with combination therapy of cetuximab and avelumab. Among 60 SCCA patients, the ORR was 17% in the combination arm compared with 10% in avelumab monotherapy cohort. In the combination arm, the median PFS was 3.9 months and the median OS was 7.8 months [27]. Bevacizumab, a vascular endothelial growth factor inhibitor (VEGFi), is shown to decrease levels of pro-angiogenic factors such as VEGF, angiostatin-1, and follistatin, and of inflammatory cytokines such as interferon γ, IL4, and IL17. In addition, it has been demonstrated to improve cytotoxic T-cell response and dendritic cell promotion within the tumor microenvironment and hence enhance the anti-tumor immune response [30]. Morris et al. reported a single-arm phase II study on the combination of atezolizumab and bevacizumab in relapsed, refractory SCCA (NCT03074513). Among 20 patients receiving the combination, the ORR was 10%, the SD was 55%, the median PFS was 4.1 months, and the median OS was 11.6 months. However, the combination resulted in 35% grade ≥ 3 adverse events (AE) (infection, hyponatremia, and hypertension). One patient experienced a grade 5 AE due to bowel perforation [31]. Both of these trials are promising but need further validation and correlative studies to obtain dependable results.

As previously mentioned, TGFβ can lead to immune evasion through an increase in recruitment of regulatory T-cells, myeloid-derived suppressor T-cells (MDCS), and NK cells, which further adds to immune evasion and tumor growth [32]. In preclinical studies, the combination of TGFβ and PD1/PD-L1 has resulted in modifying the immune evasion of the tumor cells by immune modulation from a decrease in recruitment of MDSC, T-reg, and NK cells to enhance the anti-tumor activity [33]. Bintrafusp-alpha is a first-in-class bifunctional fusion protein composed of the extracellular domain of TGFβ–βRII (a TGF-β “trap”) [33]. A phase II clinical trial of bintrafusp-alpha in patients with HPV-associated malignancies (NCT03427411) reported data on SCCA patients participating in the trial. Among the six patients with advanced-stage SCCA, the confirmed ORR was 30.5%, eight patients had stable disease with a DCR of 44.1%. A delayed response was observed in three additional patients with a total response rate of 35.6%. Among six SCCA patients, two patients achieved CR and partial response (PR), respectively. Overall, 83.1% patients experienced any AE and 27.1% patients experienced grade ≥ 3 AE [34]. A pooled analysis of these trials was reported (ESMO 2021), with data on a total of nine patients with SCCA. The ORR was seen in two of nine SCCA patients. Overall, the RR was 28% among all patients (*n* = 75). The median OS was 21.3 months [35].

EGFRis and VEGFis have shown to have immune modulator properties that could result in improved response to ICIs when used in combination, especially with EGFRis; however, this improvement in response rate is not translated to improved OS [27]. In contrast, bintrafusp-alpha, that is, a combination of PD-L1 and TGFβ, demonstrated a much better response rate and presents an exciting option for patients with SCCA.

## 7. Adoptive Cellular Therapy

Adoptive Cellular therapy is an emerging area of interest in solid tumors, including epithelial tumors such as anal cancer. Genetically engineered T-cell receptors (TCR), that is, T-cells directed against cancer antigens, have been studied in various solid tumors such as melanoma, sarcoma, and lung cancer [36]. HPV-associated epithelial cancers express E6 and E7 oncoproteins that drive the malignancy and contribute to the metastatic potential. These oncoproteins are absent from normal cells [37]. E7 could not be targeted by the traditional chimeric antigen receptor (CAR) T-cells due to intracellular location of the E7. As a result, investigators have identified high avidity TCR targeting HLA-A*02:01. These genetically engineered T-cells express TCR (E7 TCR T-cells) identify and engage HPV+ tumor cells and result in tumor regression [38,39].

In a first in-human phase I/II trial of T-cell receptor gene therapy for HPV-associated epithelial cancer (NCT02280811), autologous genetically engineered T-cells expressing high-avidity TCR directed against HLA-A*02:01-restricted E6 epitome (E6 T-cell receptor) were infused in 12 patients (treated with prior platinum-based therapy). No dose limiting toxicities were observed in the phase I portion. Two patients observed an objective response in the highest-dose cohort. One patient with lung metastases experienced complete regression of one metastasis and partial regression of two remaining metastases. A durable response of three years was observed in this patient. Four of these patients had SCCA with metastases [40]. In another similar phase I/II open-label clinical trial (NCT02858310), 12 metastatic HPV-16 positive patients received autologous genetically engineered T-cell restricted for HPV16-E7 oncoprotein (E7 TCR cells). These patients progressed on a standard of care regimen. Among these patients, two had metastatic SCCA. One of these patients experienced PR lasting for nine months. Overall, 50% of patients experienced PR and four out of eight patients had anti-PD-1 refractory disease [39]. This response rate was higher than what was observed in the NCT02280811 trial targeting E6 oncogene (50% vs. 16.7%) indicating higher antitumor functions of E7 TCR cells [40].

In NCT01585428, a phase II clinical trial, autologous-tumor-infiltrating leukocytes (TIL) were infused in patients with HPV-related metastatic malignancies. This trial included cervical and non-cervical cancer patients (*n* = 29). Five patients had SCCA. The ORR was 28% in the cervical cancer cohort and 18% (2/11) in the non-cervical cancer cohort. The one anal cancer patient with lung cancer achieved PR lasting for 4 months. HPV-reactive T-cells were higher in responder patients [41].

Cellular therapy is a novel concept and is currently an area of active research among a number of solid malignancies. E6 and E7 are the oncoproteins in HPV-related epithelial cancers and are the potential targets expressed by genetically engineered TCR. There are no anal-cancer-specific cellular therapy trials, and therefore generalizability of the reported trials is difficult at this juncture, but this data further elucidates the potential benefit of cell therapy in advanced anal cancer.

## 8. Vaccine Therapy

Cancer vaccines are another therapeutic strategy in many cancers due to their immunogenic potential. There have been numerous vaccine trials in different cancer types. Antigen selection is an important step in anti-cancer vaccine development. These antigens are recognized by T-lymphocytes mounting an antitumor immune response [42,43]. Owing to the immunogenicity of HPV-related epithelial cancers, vaccines have been investigated in HPV-related advance malignancies either alone or in combination with ICIs. Vaccines may enhance anti-cancer activity of the ICIs by activating the tumor-specific T-cells. ICIs may also enhance the immunogenicity of the vaccines and their immune response by modulating the tumor microenvironment [42]. In a phase II trial (NCT02426892), 24 patients with recurrent or metastatic HPV-16-positive tumors were administered a synthetic peptide HPV 16 vaccine (ISA101) in combination with nivolumab. The majority of these patients were oropharyngeal (22) and one patient had SCCA. The ORR was 33%, median PFS was 2.7 months, and median OS was 17.5 months [42]. The long-term follow up of this study (median follow up of 46.5 months) showed the median duration of response was 11.2 months, and three patients (38%) with objective response were free of progression at three years. The median and three-year OSs were 15.3 months (95% CI 10.6–27.2) and 12.5% (95% CI 4.3% to 36%), respectively [43].

Another approach has utilized bioengineered bacteria with a potential to enhance tumor-specific responses by manipulating the tumor microenvironment. ADXs11-00 vaccine is a live irreversibly attenuated strain of Listeria Monocytogenes, and is a vaccine that is bioengineered to secrete HPV 16 E7 oncogene, containing fusion proteins. The phagocytosis results in E7-restricted T-cell presentation, activation, and antibody production. In NCT02399813, a single-arm phase II trial, 36 patients with persistent/recurrent or previously metastatic SCCA were given ADXS11-001. The ORR was 3.4% (only one patient experienced a response) and the 6-month PFS was 15.5%. Grade 3 AEs were observed in 10 patients with the most common being cytokine-release symptoms [44].

Anti-tumor vaccine is an active area of research and has shown promise in metastatic SCCA. ISA101 + nivolumab has demonstrated a decent response rate and survival benefit; however, ADXs11-00 has a modest activity at most. Further studies, including large phase III trials, are needed to study their impact at larger scale.

The immunotherapy trials in locally advanced and/or metastatic settings are summarized in Table 2.

## 9. Conclusions and Future Directions

Squamous cell carcinomas of anal cancers are rare cancers associated with HPV 16 and 18. Immune-checkpoint-inhibitor-based therapies are becoming the mainstay of treatment in many solid tumors, including anal cancer. With increasing incidence of SCCA over the past two decades, there has been increased interest in exploring novel immunotherapies in this disease.

In non-metastatic (early-stage) SCCA, the results of trials ECOG-ACRIN 2165 [14] and RADIANCE [15] are awaited and will demonstrate the results of combining ICIs (nivoumab and durvalumab, respectively) with traditional definitive chemoradiation therapy. Additionally, there are three additional ongoing trials in the localized/locally advanced SCCA setting incorporating immunotherapy with chemoradiation: NCT04719988, NCT04046133, and NCT01671488 [45,46,47]. All of these trials utilize the principle of immunologically hot SCCA with a hope that incorporation of immunotherapy will help to improve the disease-free survival. This immunotherapeutic approach may play a pivotal role in the future of localized SCCA.

In locally advanced and/or metastatic SCCA, other novel immunotherapy targets such as anti-cytotoxic T-lymphocyte antigen 4 (CTLA4) (ipilimumab), anti-LAG3 agent (relatlimab), and anti-CD38 antibody (daratumumab) are being explored. The NCT02314169 trial is a phase II study evaluating the combination of nivolumab and ipilimumab in previously treated SCCA in metastatic settings [48]. Checkmate 358 (NCT02488759) is multicohort phase I/II trial, evaluating nivolumab as a monotherapy or in combination with ipilimumab, or relatlimab, or daratumumab in HPV-positive and HPV-negative epithelial tumors including metastatic SCCA [11].

Similarly, there are several ongoing trials investigating the combination of ICIs and cytotoxic chemotherapy. Chemotherapy may increase the effect of immunotherapy by increasing the immunogenicity such as by loss of p53, inducing immunogenic cell death, interfering myeloid derived suppressor cells (MDSC), etc. [49,50,51]. POD1UM-303/InterAACT 2 (NCT04472429) is a randomized phase III trial evaluating the role of chemotherapy carboplatin and paclitaxel with or without an immunotherapy [52].

There are several ongoing vaccine trials incorporating the E6, E7 oncoproteins either alone or in combination with various agents such as IL-12, PD-L1 (NCT03439085), and bintrafusp-alfa (NCT04432597) in recurrent metastatic HPV-associated cancers including SCCA. Human telomerase reverse transcriptase (hTERT) is expressed in 85% of human cancers and results in the pathogenesis of malignancy [53]. hTERT represents another target for the vaccination trials and is being explored in SCCA. These vaccines can help overcome immunogenic tolerance to hTERT and result in an anti-tumor response [54]. VolaTIL (NCT03946358) is a phase II trial exploring the combination of a vaccine composed of MHC-class restricted peptides (UCP2 and UCP4), which is called UCPVax, and is derived from hTERT in combination with atezolizumab in locally advanced/metastatic HPV-positive squamous cell cancers including SCCA [55]. HESITA is an ongoing phase I trial (NCT02379520) involving autologous T-cells specific to HPV 16/18 E6/E7 with or without nivolumab in HPV-positive squamous cell cancers including SCCA [56]. Due to the potential role of cellular therapy in SCCA and based on the results of NCT02399813, the work on E7 TCR is being extended to an ongoing phase II clinical trial (NCT02858310) in metastatic or recurrent/refractory HPV-16-positive patients [39].

In conclusion, immunotherapeutic approaches with novel immunotherapies are being actively explored in SCCA. We anticipate that results from some of these exciting ongoing trials in SCCA are expected to change our current practice guidelines in the few years. With the use of immunotherapy, we also recommend and anticipate identification of appropriate biomarkers to predict response in this disease.

## Figures and Tables

**Table 1:** Ongoing Immunotherapy Trials in Non-Metastatic Anal Cancer.

Ongoing Immunotherapy Trials in Non-Metastatic Anal Cancer						
NCT	Phase	N	Type of Study	Treatment Arms	Setting	End Points
NCT04230759 (RADIANCE)	II	178	Randomized parallel assignment	Arm 1: chemoradiation (mitomycin C/5-FU and radiation) Arm 2: chemoradiation + durvalumab 1500 mg every 4 weeks starting 14 days prior to chemoradiation	First line	DFS, AE, cCR, OS, LRR, and others
NCT03233711 (EA2165)	III	379	Randomized parallel assignment	All patients received standard chemoradiation Arm 1: nivolumab 480 mg IV every 4 weeks for 6 cycles Arm 2: observation	Maintenance	DFS, ORR, mAE, OS, and others

DFS: Disease-free survival; AE: adverse events, cCR: complete clinical response; OS: overall survival; LRR: loco-regional recurrence; ORR: objective response rate; mAE: major adverse events.

**Table 2:** Completed Trials in Locally Advanced/Metastatic Anal Cancer.

Trials in Locally Advanced/Metastatic Anal Cancer							
NCT No.	Phase	N	Type of Study	Treatment Arms	Targets	Line of Therapy	Key Results
NCT02314169 (Moris 2017)	II	37	Single arm	Nivolumab 3 g/kg q14 days	PD-1	2nd line and beyond	ORR 24% mPFS 4.1 m mOS 11.5 m
NCT02054806 (Ott 2017) Keynote 028	I	24 (SCCA)	Single arm	Pembrolizumab 10 mg/kg in solid tumor with PD-L1 ≥ 1% q14 days	PD-1	2nd line and beyond	ORR 17% DCR 58% mPFS 3 m
NCT02628067 (Ros Willke 2019) Keynote 158	II	112	Single arm	Pembrolizumab 200 mg q21 days	PD-1	2nd line and beyond	ORR 11% ORR 15% (CPS ≥ 1) ORR 1% (CPS < 1) mPFS 2 m
NCT03597295 (Rao 2022) POD1UM 202	II	94	Single arm	Retifanlimab 500 mg q28 days	PD-1	2nd line and beyond	ORR 13.8% DCR 48.9% mPFS 2.3 m mOS 10.1 m
NCT03944252 (Lonardi 2021) CARACAS trial	II	60	Double arm	Arm 1: avelumab 10 mg/kg q14 days (monotherapy) Arm2: avelumab 10 mg/kg q14 days + cetuximab 500 mg/m^2^	PD-L1 PD-L1+ EGFRi	2nd line and beyond	ORR: 17% vs. 10% mPFS: 3.9 vs. 2.0 m mOS: 7.8 vs. 13.9 m(ARM B vs. ARM A)
NCT03074513 (Morris 2020)	II	20 (SCCA)	Single arm	Atezolizumab 1200 mg + bevacizumab 15 mg/kg q21 days	PD-L1 + VEGF-i	2nd line and beyond	ORR 10% DCR 65% mPFS 4.1 m mOS 11.6 m
NCT03427411 NCT02517398 (Strauss 2020)	Ib and II (post-hoc analysis)	59 (6 with SCCA)	Single arm	Bintrasfusp-alfa 1200 mg q 14 days	PD-L1 + TGFβ	2nd line and beyond	ORR 30.5% DCR 44.1% mPFS 2.8 m mOS NR (mOS 21.2 months in long-term follow up)
NCT02280811 (Doran 2019)	I/II	12 (4 patients with SCCA)	Single arm	HPV E6 T-cell receptor (TCR), followed by high dose aldesleukin	E6 T-cell receptor	2nd line and beyond	No DLT MTD 105 × 10^9^ E6 TCT T-cells ORR 16.7%
NCT02858310 (Nagarseth 2021)	I/II	12 (2 patients with SCCA)	Single arm	HPV E7 T-cell receptor (TCR), followed by high dose aldesleukin	E7 T-cell receptors	2nd line and beyond	MDT: 100 billion E7 TCR T-cells ORR 50%
NCT01585428 Stevanovi 2019	II	29 (5 SCCA patients)	Single arm	Young tumor-infiltrating lymphocytes (TIL) plus high dose IV aldesleukin.		2nd line and beyond	ORR 28% in cervical cohort ORR 18% in non-cervical cohort
NCT02426892 (Massarelli 2019)	II	24 (1 with SCCA)	Single arm	HPV 16 vaccination (ISA 101) SC at 100 mcg × 3 doses q 3 to 4 weeks + Nivolumab 3 mg/kg q 2 weeks beginning on day 8 after the first vaccine dose.	PD-1 + HPV 16 peptide vaccine	2nd line and beyond	ORR 33% mPFS 2.7 m mOS 17.5 m
NCT02399813 (Eng 2020)	II	36	Single arm	axalimogene filolisbac (ADXS11-001) q 3 weeks at a dose of 1 × 10^9^ cfu	HPV 16 E7 protein	2nd line and beyond	ORR was 3.4% 6-month PFS was 15.5%

ORR: objective response rate; PFS: progression-free survival; OS: overall survival; DCR: disease control rate; SCCA: squamous cell carcinoma of the anus; PD-1: programmed cell death protein 1; PD-L1: programmed death ligand-1; EGFRi: epidermal growth factor inhibitor; VEGF-I vascular endothelial growth factor inhibitor; TGFβ: transforming growth factor beta; HPV: human papilloma virus.

## References

[1] Siegel R.L., Miller K.D., Wagle N.S., Jemal A. (2023). Cancer statistics, 2023. CA Cancer J. Clin..

[2] (2023). Cancer Facts & Figures. https://www.cancer.org/research/cancer-facts-statistics/all-cancer-facts-figures/2023-cancer-facts-figures.html.

[3] Deshmukh A.A., Suk R., Shiels M.S., Sonawane K., Nyitray A.G., Liu Y., Gaisa M.M., Palefsky J.M., Sigel K. (2020). Recent Trends in Squamous Cell Carcinoma of the Anus Incidence and Mortality in the United States, 2001-2015. J. Natl. Cancer Inst..

[4] SEER * Explorer: An Interactive Website for SEER Cancer Statistics. https://seer.cancer.gov/explorer/.

[5] Daling J.R., Madeleine M.M., Johnson L.G., Schwartz S.M., Shera K.A., Wurscher M.A., Carter J.J., Porter P.L., Galloway D.A., McDougall J.K. (2004). Human papillomavirus, smoking, and sexual practices in the etiology of anal cancer. Cancer.

[6] Clifford G.M., Georges D., Shiels M.S., Engels E.A., Albuquerque A., Poynten I.M., de Pokomandy A., Easson A.M., Stier E.A. (2021). A meta-analysis of anal cancer incidence by risk group: Toward a unified anal cancer risk scale. Int. J. Cancer.

[7] Frisch M., Glimelius B., Wohlfahrt J., Adami H.O., Melbye M. (1999). Tobacco smoking as a risk factor in anal carcinoma: An antiestrogenic mechanism?. J. Natl. Cancer Inst..

[8] Carr R.M., Jin Z., Hubbard J. (2021). Research on Anal Squamous Cell Carcinoma: Systemic Therapy Strategies for Anal Cancer. Cancers.

[9] Colón-López V., Shiels M.S., Machin M., Ortiz A.P., Strickler H., Castle P.E., Pfeiffer R.M., Engels E.A. (2018). Anal Cancer Risk Among People with HIV Infection in the United States. J. Clin. Oncol..

[10] Morel A., Neuzillet C., Wack M., Lameiras S., Vacher S., Deloger M., Servant N., Veyer D., Péré H., Mariani O. (2019). Mechanistic Signatures of Human Papillomavirus Insertions in Anal Squamous Cell Carcinomas. Cancers.

[11] Ciardiello D., Guerrera L.P., Maiorano B.A., Parente P., Latiano T.P., Di Maio M., Ciardiello F., Troiani T., Martinelli E., Maiello E. (2022). Immunotherapy in advanced anal cancer: Is the beginning of a new era?. Cancer Treat. Rev..

[12] Ott P.A., Piha-Paul S.A., Munster P., Pishvaian M.J., van Brummelen E.M.J., Cohen R.B., Gomez-Roca C., Ejadi S., Stein M., Chan E. (2017). Safety and antitumor activity of the anti-PD-1 antibody pembrolizumab in patients with recurrent carcinoma of the anal canal. Ann. Oncol..

[13] Smola S., Trimble C., Stern P.L. (2017). Human papillomavirus-driven immune deviation: Challenge and novel opportunity for immunotherapy. Ther. Adv. Vaccines.

[14] NCT03233711 Nivolumab after Combined Modality Therapy in Treating Patients with High Risk Stage II-IIIB Anal Cancer. NCT03233711.

[15] Martin D., Balermpas P., Gollrad J., Weiß C., Valentini C., Stuschke M., Schäfer H., Henckenberens C., Debus J., Krug D. (2020). RADIANCE-Radiochemotherapy with or without Durvalumab in the treatment of anal squamous cell carcinoma: A randomized multicenter phase II trial. Clin. Transl. Radiat. Oncol..

[16] Eng C. (2006). Anal cancer: Current and future methodology. Cancer Investig..

[17] Ajani J.A., Carrasco C.H., Jackson D.E., Wallace S. (1989). Combination of cisplatin plus fluoropyrimidine chemotherapy effective against liver metastases from carcinoma of the anal canal. Am. J. Med..

[18] Faivre C., Rougier P., Ducreux M., Mitry E., Lusinchi A., Lasser P., Elias D., Eschwege F. (1999). 5-Fluorouracile and cisplatinum combination chemotherapy for metastatic squamous-cell anal cancer. Bull. Cancer.

[19] Kim S., Jary M., Mansi L., Benzidane B., Cazorla A., Demarchi M., Nguyen T., Kaliski A., Delabrousse E., Bonnetain F. (2013). DCF (docetaxel, cisplatin and 5-fluorouracil) chemotherapy is a promising treatment for recurrent advanced squamous cell anal carcinoma. Ann. Oncol..

[20] Hainsworth J.D., Burris H.A., Meluch A.A., Baker M.N., Morrissey L.H., Greco F.A. (2001). Paclitaxel, carboplatin, and long-term continuous infusion of 5-fluorouracil in the treatment of advanced squamous and other selected carcinomas: Results of a Phase II trial. Cancer.

[21] Jaiyesimi I.A., Pazdur R. (1993). Cisplatin and 5-fluorouracil as salvage therapy for recurrent metastatic squamous cell carcinoma of the anal canal. Am. J. Clin. Oncol..

[22] Rao S., Sclafani F., Eng C., Adams R.A., Guren M.G., Sebag-Montefiore D., Benson A., Bryant A., Peckitt C., Segelov E. (2020). International Rare Cancers Initiative Multicenter Randomized Phase II Trial of Cisplatin and Fluorouracil Versus Carboplatin and Paclitaxel in Advanced Anal Cancer: InterAAct. J. Clin. Oncol..

[23] Morris V.K., Salem M.E., Nimeiri H., Iqbal S., Singh P., Ciombor K., Polite B., Deming D., Chan E., Wade J.L. (2017). Nivolumab for previously treated unresectable metastatic anal cancer (NCI9673): A multicentre, single-arm, phase 2 study. Lancet Oncol..

[24] Chung H.C., Ros W., Delord J.P., Perets R., Italiano A., Shapira-Frommer R., Manzuk L., Piha-Paul S.A., Xu L., Zeigenfuss S. (2019). Efficacy and Safety of Pembrolizumab in Previously Treated Advanced Cervical Cancer: Results from the Phase II KEYNOTE-158 Study. J. Clin. Oncol..

[25] Marabelle A., Cassier P.A., Fakih M., Kao S., Nielsen D., Italiano A., Guren T.K., van Dongen M.G.J., Spencer K., Bariani G.M. (2022). Pembrolizumab for previously treated advanced anal squamous cell carcinoma: Results from the non-randomised, multicohort, multicentre, phase 2 KEYNOTE-158 study. Lancet Gastroenterol. Hepatol..

[26] Rao S., Anandappa G., Capdevila J., Dahan L., Evesque L., Kim S., Saunders M.P., Gilbert D.C., Jensen L.H., Samalin E. (2022). A phase II study of retifanlimab (INCMGA00012) in patients with squamous carcinoma of the anal canal who have progressed following platinum-based chemotherapy (POD1UM-202). ESMO Open.

[27] Lonardi S., Prete A.A., Morano F., Messina M., Formica V., Corsi D.C., Orciuolo C., Frassineti G.L., Zampino M.G., Casagrande M. (2021). Randomized phase II trial of avelumab alone or in combination with cetuximab for patients with previously treated, locally advanced, or metastatic squamous cell anal carcinoma: The CARACAS study. J. Immunother. Cancer.

[28] Wang L., Wei Y., Fang W., Lu C., Chen J., Cui G., Diao H. (2017). Cetuximab Enhanced the Cytotoxic Activity of Immune Cels during Treatment of Colorectal Cancer. Cell. Physiol. Biochem..

[29] Fasano M., Della Corte C.M., Di Liello R., Barra G., Sparano F., Viscardi G., Iacovino M.L., Paragliola F., Famiglietti V., Ciaramella V. (2020). Induction of natural killer antibody-dependent cell cytotoxicity and of clinical activity of cetuximab plus avelumab in non-small cell lung cancer. ESMO Open.

[30] Martino E.C., Misso G., Pastina P., Costantini S., Vanni F., Gandolfo C., Botta C., Capone F., Lombardi A., Pirtoli L. (2016). Immune-modulating effects of bevacizumab in metastatic non-small-cell lung cancer patients. Cell Death Discov..

[31] Morris V., Liu S., Johnson B., Prasad S., Mahvash A., Bhosale P., Rubin M.L., Rothschild N., Futreal A., Wistuba I.I. (2020). 403MO Atezolizumab in combination with bevacizumab for patients with unresectable/metastatic anal cancer. Ann. Oncol..

[32] Ravi R., Noonan K.A., Pham V., Bedi R., Zhavoronkov A., Ozerov I.V., Makarev E., Artemov A.V., Wysocki P.T., Mehra R. (2018). Bifunctional immune checkpoint-targeted antibody-ligand traps that simultaneously disable TGFβ enhance the efficacy of cancer immunotherapy. Nat. Commun..

[33] Lan Y., Zhang D., Xu C., Hance K.W., Marelli B., Qi J., Yu H., Qin G., Sircar A., Hernández V.M. (2018). Enhanced preclinical antitumor activity of M7824, a bifunctional fusion protein simultaneously targeting PD-L1 and TGF-β. Sci. Transl. Med..

[34] Strauss J., Gatti-Mays M.E., Cho B.C., Hill A., Salas S., McClay E., Redman J.M., Sater H.A., Donahue R.N., Jochems C. (2020). Bintrafusp alfa, a bifunctional fusion protein targeting TGF-β and PD-L1, in patients with human papillomavirus-associated malignancies. J. Immunother. Cancer.

[35] Strauss J., Gatti-Mays M.E., Cho B.C., Hill A., Salas S., McClay E., Redman J.M., Sater H.A., Donahue R.N., Jochems C. Long-term follow-up of patients with human papillomavirus (HPV)–associated malignancies treated with bintrafusp alfa, a bifunctional fusion protein targeting TGF-β and PD-L1. ESMO Congr..

[36] Robbins P.F., Morgan R.A., Feldman S.A., Yang J.C., Sherry R.M., Dudley M.E., Wunderlich J.R., Nahvi A.V., Helman L.J., Mackall C.L. (2011). Tumor regression in patients with metastatic synovial cell sarcoma and melanoma using genetically engineered lymphocytes reactive with NY-ESO-1. J. Clin. Oncol..

[37] Trimble C.L., Frazer I.H. (2009). Development of therapeutic HPV vaccines. Lancet Oncol..

[38] Jin B.Y., Campbell T.E., Draper L.M., Stevanović S., Weissbrich B., Yu Z., Restifo N.P., Rosenberg S.A., Trimble C.L., Hinrichs C.S. (2018). Engineered T cells targeting E7 mediate regression of human papillomavirus cancers in a murine model. JCI Insight.

[39] Nagarsheth N.B., Norberg S.M., Sinkoe A.L., Adhikary S., Meyer T.J., Lack J.B., Warner A.C., Schweitzer C., Doran S.L., Korrapati S. (2021). TCR-engineered T cells targeting E7 for patients with metastatic HPV-associated epithelial cancers. Nat. Med..

[40] Doran S.L., Stevanović S., Adhikary S., Gartner J.J., Jia L., Kwong M.L., Faquin W.C., Hewitt S.M., Sherry R.M., Yang J.C. (2019). T-cell receptor gene therapy for human papillomavirus–associated epithelial cancers: A first-in-human, phase I/II study. J. Clin. Oncol..

[41] Stevanović S., Helman S.R., Wunderlich J.R., Langhan M.M., Doran S.L., Kwong M.L., Somerville R.P., Klebanoff C., Kammula U.S., Sherry R.M. (2019). A phase II study of tumor-infiltrating lymphocyte therapy for human papillomavirus–associated epithelial cancers. Clin. Cancer Res..

[42] Massarelli E., William W., Johnson F., Kies M., Ferrarotto R., Guo M., Feng L., Lee J.J., Tran H., Kim Y.U. (2019). Combining immune checkpoint blockade and tumor-specific vaccine for patients with incurable human papillomavirus 16–related cancer: A phase 2 clinical trial. JAMA Oncol..

[43] De Sousa L.G., Rajapakshe K., Canales J.R., Chin R.L., Feng L., Wang Q., Barrese T.Z., Massarelli E., William W., Johnson F.M. (2022). ISA101 and nivolumab for HPV-16+ cancer: Updated clinical efficacy and immune correlates of response. J. Immunother. Cancer.

[44] Eng C., Fakih M., Amin M., Morris V., Hochster H.S., Boland P.M., Uronis H. (2020). A phase II study of axalimogene filolisbac for patients with previously treated, unresectable, persistent/recurrent loco-regional or metastatic anal cancer. Oncotarget.

[45] NCT04719988 Anti-PD-1 and mDCF Followed by Chemoradiotherapy in Patients with Stage III Squamous Cell Anal Carcinoma. (INTERACT-ION). NCT04719988.

[46] NCT04046133 Phase 1b/II Trial of Pembrolizumab Plus IMRT in Stage III/IV Carcinoma of Anus (CORINTH). NCT04046133.

[47] NCT01671488 A Phase I/II Evaluation of ADXS11-001, Mitomycin, 5-fluorouracil (5-FU) and IMRT for Anal Cancer (276). NCT01671488.

[48] Morris V.K., Eng C. (2018). Role of Immunotherapy in the Treatment of Squamous Cell Carcinoma of the Anal Canal. J. Natl. Compr. Canc. Netw..

[49] Kim S., Jary M., André T., Vendrely V., Buecher B., François E., Bidard F.C., Dumont S., Samalin E., Peiffert D. (2017). Docetaxel, cisplatin, and 5-fluorouracil (DCF) chemotherapy in the treatment of metastatic or unresectable locally recurrent anal squamous cell carcinoma: A phase II study of French interdisciplinary GERCOR and FFCD groups (Epitopes-HPV02 study). BMC Cancer.

[50] Wahl A.F., Donaldson K.L., Faircnild C., Lee F.Y., Foster S.A., Demers G.W., Galloway D.A. (1996). Loss of normal p53 function confers sensitization to Taxol by increasing G2/M arrest and apoptosis. Nat. Med..

[51] Kim S., Buecher B., André T., Jary M., Bidard F.-C., Ghiringhelli F., François-Clément B., Taieb J., Smith D., De La Fouchardière C. (2020). Atezolizumab plus modified docetaxel-cisplatin-5-fluorouracil (mDCF) regimen versus mDCF in patients with metastatic or unresectable locally advanced recurrent anal squamous cell carcinoma: A randomized, non-comparative phase II SCARCE GERCOR trial. BMC Cancer.

[52] NCT04444921 EA2176: Phase 3 Clinical Trial of Carboplatin and Paclitaxel +/− Nivolumab in Metastatic Anal Cancer Patients. NCT04444921.

[53] Collins K., Mitchell J.R. (2002). Telomerase in the human organism. Oncogene.

[54] Thalmensi J., Pliquet E., Liard C., Escande M., Bestetti T., Julithe M., Kostrzak A., Pailhes-Jimenez A.S., Bourges E., Loustau M. (2016). Anticancer DNA vaccine based on human telomerase reverse transcriptase generates a strong and specific T cell immune response. Oncoimmunology.

[55] Rebucci-Peixoto M., Vienot A., Adotevi O., Jacquin M., Ghiringhelli F., De La Fouchardiere C., You B., Maurina T., Kalbacher E., Bazan F. (2022). A Phase II Study Evaluating the Interest to Combine UCPVax, a Telomerase CD4 TH1-Inducer Cancer Vaccine, and Atezolizumab for the Treatment of HPV Positive Cancers: VolATIL Study. Front. Oncol..

[56] NCT02379520 HPV-16/18 E6/E7-Specific T Lymphocytes, Relapsed HPV-Associated Cancers, HESTIA (HESTIA). NCT02379520.

